# Fish oil and probiotics supplementation through milk chocolate improves spatial learning and memory in male Wistar rats

**DOI:** 10.3389/fnut.2022.1023653

**Published:** 2022-11-17

**Authors:** Paulinna Faccinetto-Beltrán, Luis Octavio Aguirre-López, Jacinto Bañuelos-Pineda, Edwin E. Reza-Zaldívar, Arlette Santacruz, Carmen Hernández-Brenes, Esther Pérez-Carrillo, Daniel A. Jacobo-Velázquez

**Affiliations:** ^1^Tecnologico de Monterrey, The Institute for Obesity Research, Zapopan, Jal, Mexico; ^2^Tecnologico de Monterrey, Escuela de Ingeniería y Ciencias, Zapopan, Mexico; ^3^Laboratorio de Morfofisiología, Departamento de Medicina Veterinaria, Centro Universitario de Ciencias Biológicas y Agropecuarias, Universidad de Guadalajara, Zapopan, Mexico; ^4^Tecnologico de Monterrey, Escuela de Ingeniería y Ciencias, Monterrey, Mexico; ^5^Tecnologico de Monterrey, The Institute for Obesity Research, Monterrey, Mexico

**Keywords:** spatial learning and memory, cognitive capacity, milk chocolate, omega-3 polyunsaturated fatty acids, Barnes maze test, gut-brain axis

## Abstract

**Background:**

Cognition and brain function is critical through childhood and should be improved with balanced diets. Incorporating bioactive ingredients such as omega-3 polyunsaturated fatty acids (ω3 PUFAs) and probiotics into food formulations could be used as an approach to improve cognitive function. This study evaluated the effects on cognitive capacity of complementing rodent diets with chocolate, by itself and in combination with ω3 PUFAs from fish oil and probiotics.

**Methods:**

Spatial learning and memory in the rats were determined by the Barnes maze test in short- and long-term memory. Samples from the cecum were obtained to assess microbial counts (*Lactobacillus, Bifidobacterium, Enterobacteriaceae*, and total bacteria), and brains were recovered to analyze the neural morphology of the tissues. Also, glucose, brain weights, and epididymal tissue were analyzed.

**Results:**

The combination of chocolate with fish oil and probiotics improved the memory of rats compared to the result of each bioactive compound when evaluated separately. Treatments did not affect sugar level, epididymal adipose tissue, or brain weight. On the other hand, consuming probiotics alone or in combination with chocolate decreased *Enterobacteria* counts, while *Lactobacillus* and *Bifidobacteria* counts were not affected. Neural morphological analysis showed that combining chocolate with probiotics and ω3 PUFAs increased the number of neurons in the hippocampal CA1 and CA3 regions.

**Conclusion:**

Chocolate added with probiotics and ω3 PUFAs improved spatial memory and learning in the studied model.

## Introduction

Cognitive development refers to the level at which a person acquires and processes information, including learning and solving problems (1). Nowadays, a big concern is to enhance cognitive development through childhood to decrease the risk of unwanted adult outcomes (2). Childhood is critical because children are vulnerable to low-calorie diets that affect brain functions (3). It is also an important stage for cognitive development because it can predict future characteristics such as academic achievement, success, and how they fit into society (1).

A link between balanced diets and brain development has been established because essential compounds found in food are critical to brain and cognitive development. Omega-3 polyunsaturated fatty acids (ω3 PUFAs), like docosahexaenoic acid (DHA), are the most abundant fatty acid in the central nervous system, increasing during development and are sensitive to alterations in dietary intake. A correlation has been found between low DHA levels in the blood and the increased risk of poor neural and visual development in children (4). Likewise, a relationship between higher cognition, peripheral functions, and gastrointestinal homeostasis has been reported; crosstalk denominated the gut-brain axis. In this context, diet supplementation with probiotics in the early stages can reduce the risk of neurodevelopmental disorders (5). Strains such as *Lactobacillus plantarum* 299v and *Lactobacillus rhamnosus* GG have improved cognition in children with communication disorders and patients with major depression (6, 7). Also, *L. plantarum* has been found to delay neurodegeneration in Alzheimer’s disease-induced rat model and inhibit the neurodegenerative process of the Parkinson’s disease model (8, 9). On the other hand, *L. rhamnosus* has improved cognitive function and decreased anxiety/depression behaviors (10, 11). Both ω3 PUFAs and probiotics are considered bioactive ingredients and are widely used in the food industry.

Incorporating bioactive compounds into foods to produce functional foods that fulfill consumer requirements is an excellent option to improve human health (12). Despite the benefits of functional foods, consumer acceptance depends on the type of vehicle used as the delivery system of the bioactive compounds (13). In this context, milk chocolate can be employed as a vehicle for easy administration of compounds because of its consumer acceptance and suitability for carrying both ω3 PUFAs and probiotics (14–17). In our previous study, we developed a milk chocolate formulation containing probiotics (*L. plantarum* 299v and *L. rhamnosus* GG) and fish oil with high levels of ω3 PUFAs (14). The consumer’s acceptability and physicochemical properties of chocolates added with ω3 PUFAs (at 76.0 ± 5.2 mg or 195.8 ± 6.5 mg per serving size) and probiotics were characterized. It was learned that chocolate with probiotics >1 × 10^6^ CFU and 76.0 ± 5.2 mg of ω3 PUFAs per serving size (12 g) showed adequate consumer acceptability and physiochemical properties similar to the control chocolate without bioactive ingredients (12). However, further research is needed to validate the effect of the developed chocolate on cognitive development.

Although it has been reported that probiotics and fish oil contribute to cognitive development, their effects have not been previously validated using chocolate as a delivery system. Due to the high acceptability of chocolate by children, it could be an adequate alternative to capsules or dietary supplements for the delivery of these bioactive ingredients. Therefore, in the present study, chocolate was used as a delivery system of ω3 PUFAs and probiotics and provided to male Wistar rats to determine the effects of the functional chocolate on the cognitive abilities, brain development, and gut-microbiota.

## Materials and methods

### Reagents and active ingredients

The chocolate bars were elaborated with milk-chocolate pellets of 33% cocoa (Vanleer, Barry Callebaut, Zurich, CH). The fish oil (Omega Pure^®^) was purchased from America Alimentos S.A. de C.V. (Zapopan, Jalisco, MX). Probiotic strains *Lactobacillus plantarum* 299v (DSMZ 9843) and *Lactobacillus rhamnosus* GG (ATCC 7469) were obtained from the German Collection of Microorganisms and Cell Cultures (DSMZ, Braunschweig, DE) and American Type Culture Collection (ATCC, Manassas, VA, US), respectively. Sodium alginate was purchased from DEIMAN (CDMX, MX) and maltodextrin food grade from Best Ingredients (Monterrey, NL, MX). MRS agar was obtained from BD Difco*™* (NJ, US). For the euthanasia of rats, a mix of Ketamine (Boehringer Ingelheim, JC, MX) and Xylazine (PROCIN^®^, PiSA, JC, MX.) were utilized.

### Manufacturing of milk-chocolate added with fish oil rich in omega-3 polyunsaturated fatty acids and probiotics

Two vehicles were considered to evaluate the effect of fish oil (FO) rich in ω3 PUFAs and probiotics on rats’ cognitive abilities and neural development: milk chocolate and commercial rodent food (Purina Rodent Chow^®^ pellets, CDMX, México). Milk chocolate added with ω3 PUFAs and probiotics was manufactured according to the procedure described by Faccinetto-Beltrán et al. (14). Probiotics were microencapsulated with a mix of maltodextrin (10% *w*/*v*) and sodium alginate (2% *w*/*v*) using a spray-dryer (ADL 311S, Yamato Scientific Co., Ltd., Santa Clara, CA, USA) (14). Chocolate pellets of 33% cocoa were fully melted and homogenized in a water bath at 43°C in a stainless-steel container. Then, the chocolate was tempered to 29°C using a marble plate. Afterward, the chocolate was transferred to a stainless-steel container and returned to the water bath until reaching 31°C. Fish oil containing EPA (13.8%) and DHA (11.8%) was used as the source of ω3 PUFAs, which, together with the microencapsulated probiotics mix, were added during the tempering process according to each treatment. Fish oil was added to the chocolate formulation at 3.24%. At the end of the manufacturing process, chocolates with >1 × 10^6^ CFU of probiotics and/or 76 mg of ω3 PUFAs per portion (12 g) were obtained. The physicochemical properties and consumer acceptability of the functional chocolate under evaluation were previously reported (14).

Commercial rodent food-based pellet treatments were prepared to simulate the usual rat’s diet by mixing 30 g of milled aliment and 30 g of distilled water until a mass formation. Then FO and probiotics were added and homogenized to form thin rolls according to each treatment. The formulations were dehydrated in the oven at 45°C for 6 h. Finally, UV light treatment was applied for 15 min in a laminar flow hood to eliminate possible contaminants. As previously mentioned in the chocolate formulation section, the powder containing microencapsulated probiotics and FO was added to obtain >1 × 10^6^ CFU of probiotics per portion and/or 76 mg of ω3 PUFAs per portion. A total of eight treatments were formulated, as shown in Table 1.

**TABLE 1:** Treatments composition of rat diets.

Treatment^1^	Vehicle		Active ingredients	
	Commercial rodent food	Chocolate	Probiotics mixture	Fish oil (3.24%)
Control	x			
Prob	x		x	
FO	x			x
Prob + FO	x		x	x
Chocolate		x		
Chocolate + Prob		x	x	
Chocolate + FO		x		x
Chocolate + Prob + FO		x	x	x

^1^Control, commercial rodent food-based diet alone; FO, fish oil; Prob, probiotics.

### Experimental model and diet administration

The 64 post-weaning male Wistar rats (50–100 g) used in this study were purchased from Circulo ADN (Mexico City, México). A total of eight treatments (*n* = 8) were evaluated as indicated in Table 1, considering the two vehicles: milk chocolate and commercial rodent food (Purina Rodent Chow^®^ pellets, CDMX, México), and two active ingredients (probiotics and FO): (1) control, (2) Probiotics (Prob), (3) Fish oil (FO), (4) Prob + FO, (5) chocolate, (6) chocolate + Prob, (7) chocolate + FO, and (8) chocolate + Prob + FO.

**FIGURE 1:**
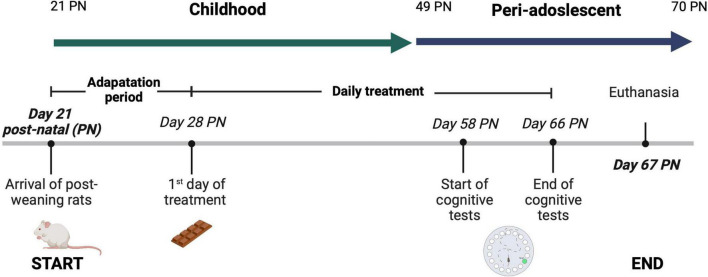
Experimental protocol. Treatments were administered for 38 days, starting after the seven-day adaptation period until the previous day of sacrifice, considering the seven-day cognition test. Each rat group was given its respective treatment from 2 p.m. to 5 p.m. After 31 days of treatment (day 58 post-natal (PN), the rats were subjected to the Barnes Maze (BM) test to evaluate spatial learning and short and long-term memory. The rats were kept under standard nursery conditions at a controlled temperature of 22°C ± 1°C, with light-dark cycles of 12 h, allowing *ad libitum* access to water and commercial rodent food throughout the experimental period. Body weights were recorded weekly. A 38-day administration period was selected since is the required period to observe an effect on rat’s behavior when probiotics are supplemented (18). Also, the supplementation of the treatment started at the post-weaning period, 21 days post-natal (PN), because is the age at which the hippocampus and pre-frontal cortex begin to contribute to learning processes (19). Rats have an accelerated childhood compared to humans, and this investigation is targeted to assess the effect of childhood supplementation of nutraceuticals on spatial learning and memory in male Wistar rats. Therefore, in this protocol the animal model was supplemented with the bioactive ingredients during childhood and no longer than adulthood (>70 post-natal) (20). This experimental protocol was reviewed and approved by the Research Ethics Committee of University of Guadalajara (CIN/034/2022). Figure created with BioRender.com.

For both vehicles, the treatment portion was calculated considering a daily consumption of 76 mg of ω3 per infant. The daily amount was calculated based on the serving size of 12 g of milk chocolate per 20 kg of average weight for Mexican infant. Therefore, sample portions of 0.12 ± 0.01 g were established considering a 200 g rat average weight. The chocolate tested contains the maximum amount of fish oil that could be added before affecting the acceptability of the product by consumers (14). The experimental protocol is summarized in Figure 1. Treatments were administered for 38 days, starting after the seven-day adaptation period until the previous day of sacrifice, considering the seven-day cognition test. Both bioactive ingredients (probiotics and fish oil) were stable during the 38 days of execution of the protocol (data not shown).

A 38-day administration period was selected since previous studies have shown that a 2–4 weeks supplementation is needed to positively affect the rat’s behavior when probiotics are supplemented (18). Also, the supplementation of the treatment started at the post-weaning period, 21 days post-natal (PN), because is the age at which the hippocampus and pre-frontal cortex begin to contribute to learning processes (19). In addition, rats have an accelerated childhood compared to humans, and this investigation is targeted to assess the effect of childhood supplementation of nutraceuticals on their development. Therefore, in this protocol rats were supplemented with the bioactive ingredients during childhood and no longer than adulthood (>70 post-natal) (20).

Each rat group was given its respective treatment from 2 p.m. to 5 p.m. The rats were kept under standard nursery conditions at a controlled temperature of 22°C ± 1°C, with light-dark cycles of 12 h, allowing *ad libitum* access to water and commercial rodent food throughout the experimental period. Body weights were recorded weekly. This experimental protocol was reviewed and approved by the Research Ethics Committee of Universidad de Guadalajara (CIN/034/2022).

### Memory evaluation

After 31 days of treatment, the rats were subjected to the Barnes Maze (BM) test to evaluate spatial learning and short and long-term memory, according to Barnes et al. (21). Briefly, this consisted of a black circular platform with a diameter of 120 cm raised 91 cm. In the platform’s periphery, 20 holes of 5 cm diameter were spaced by 8.5 cm between each. In the lower part of one of the holes was located a scape box (11 cm x 11 cm x 40 cm) made of acrylic material. Navigation cues were placed around the room (yellow square, pink circle, orange heart, and blue rhombus) to indicate the scape hole (21).

The test lasted seven days, during which supplementation of treatments continued. On the first day, rats underwent a habituation test phase where individually, they were placed inside a plastic cylinder (start tube) in the platform’s center for 10 s. When the time elapsed, the rat was led to the escape box, which remained for 1 min. Once the test was completed, it was transported to its housing box. All rats were randomly assessed to avoid data interference. At the end of the habituation period, the test was repeated, but the rat was allowed to freely explore the platform for 3 min. If the rat did not find the escape box at the end of the time, the rat was guided to it and kept inside for 1 min. The rats were returned to their housing boxes after the test. BM test paradigm was structured by two sessions per day for four days, where short-term memory (STM) was analyzed. The time between sessions was 45 min. Long-term memory (LTM) was determined with a single test that was performed 72 h after the fourth day of examinations. The platform, escape box, and start tube were carefully cleaned with 70% ethanol at the end of each test to avoid the influence of aromatic signals. All tests were recorded with a digital camera (Logitech C505-2012, Lausana, CH) for later analysis using the Kinovea v.0.8.15 (2011) software. In each trial, the following parameters were evaluated: total latency (s), the latency of the first exploration, latency in the escape zone, latency of the first exploration in the escape hole, and total number of errors (considering error when the rat introduced its nose into a different hole than the escape box), number of errors in the escape zone and total distance traveled (cm).

### Glucose and tissue analysis

Once the behavioral test was finished, rats were administered 80 mg/kg of Ketamine 10% and anesthetized with a cocktail using 10 mg/kg of Xylazine. Euthanasia was performed by direct pericardial puncture, and direct blood was extracted with a vacutainer. Blood stored in the vacutainer was centrifuged at 5,000 x *g* (IEC, Delhi, IN), and plasma was retrieved in 1.5 ml Eppendorf (Sigma-Aldrich, San Luis, US) tubes stored at −80°C. Using whole blood, glucose was analyzed with a blood glucose meter system and strips (Accu-Chek Performa, Hoffmann-La Roche, Basilea, CH). Cecum and the final portion of the large intestine containing fecal samples were dissected and stored at −80°C. Epididymal adipose tissue, previously separated from epididymis and testes, was weighed with Traveler portable balance TA301 (Ohaus, CDMX, México). After weighing adipose tissue, it was discarded. Briefly, the brain was extracted and separated from the cerebellum. Brain tissue was weighted and stored at −80°C in 30 ml of formaldehyde (10%).

### Quantitative real-time PCR microbial quantification

Fecal samples from each rat were collected from the cecum portion of the intestine. The cecum was surgically removed and stored at −80°C until use. Briefly, 1 g (wet weight) of feces was diluted in 9 ml of PBS (1X) and vortexed for 5 min. Afterward, 1.8 ml of the previously homogenized sample was centrifugated (3,134 × *g*, 1 min) in 2 ml vials to remove large particles. The supernatant was used for DNA extraction using the DNAeasy Ultra Clean Microbial kit (Qiagen, Hilden, Germany), following the manufacturer’s instructions. Quantitative real-time PCR (qPCR) was used to quantify *Lactobacillus*, *Bifidobacterium*, *Enterobacteriaceae*, and total bacterial count using specific primers for each microbial group (Supplementary Table 1; 22–25). PCR amplification and detection were performed with a thermal cycler Rotor gene RG3000 (Corbett Research, Sydney, AU) using an SYBR Green PCR kit (Qiagen, Hilden, GE). Each reaction mixture of 25 μl was composed of 1.25 μl for both primers (10 μM), 12.5 μl of SYBR Green I Master Mix (Qiagen, Hilden, GE), and 3 μl of DNA (5 ng/μl final concentration). Bacterial concentrations were calculated as previously described (24) using standard curves for each bacterial group.

### Neural morphological analysis in CA1 and CA3 regions of the hippocampus

#### Histological analysis

After euthanasia, rats were decapitated, and their brains were removed and fixed with 4% paraformaldehyde (pH 7.4). The brains were washed with phosphate buffer 0.01 mM for histological sectioning and subsequently dehydrated with alcohols in increasing concentrations (26). Then, brains were diaphanized and embedded in paraffin. All brains were cut into 10-μm-thick coronal sections using a rotary microtome (Leica). The analysis region was determined using the stereotaxic atlas of Paxinos and Watson (27)–from interaural 6.2 mm, bregma 2.8 mm to interaural 3.2 mm, bregma 5.8 mm. Hematoxylin and eosin staining was performed according to the protocol proposed by Fischer et al. (26).

From the sections obtained, descriptive histological analysis and quantification of the number of nuclei per field and the nuclear area of the neurons of the CA1 and CA3 regions of the hippocampus were performed using a light microscope (Leica^®^ DMLB) connected to a camera (Sony IMX582 Exmor RS, 48 megapixels, aperture f/1.79) with a 40x objective. Sixty images per group of each region of the hippocampus (30 images per hemisphere) were digitized, choosing the digitized sections of each animal at random. To analyze the number of neuronal nuclei per field, quadrants of 100 square microns were established on the digitized image. Three were chosen, and the number of nuclei within the selected area was quantified. Those identifiable neuron nuclei with complete circumference, differentiated chromatin, and compact nucleoli were taken as inclusion criteria, and as exclusion criteria of neuron nuclei with not well-defined circumference, undifferentiated chromatin and positioned on the lines of the quadrants. The nuclear area was quantified from the same images, from which 120 nuclei were selected per group to calculate the area of each chosen object subsequently. Both calibration and image analysis were performed using Motic ImagesPlus^®^ 20 ML software.

### Statistical analysis

Statistical analyses were performed with data obtained from eight replicates of each treatment. All data represent the mean values of eight replicates and their standard error. Significant differences between mean values from the BM test, qPCR microbial quantification, and glucose and tissue analyses were determined by factorial analysis of variance, followed by an LSD test (*p* < 0.05), using the JMP statistical software 14.1.0 version (SAS Institute Inc., Cart, NC, US). Neuromorphological statistical data analysis was also performed using factorial analysis of variance with a significance level of 5%. If significant differences were found, the comparison between groups were made using the Bonferroni *post-hoc* test.

## Results

### Cognitive evaluation

#### Introduction of chocolate and fish oil in the rodent diet decreased latency times in various sessions

**FIGURE 2:**
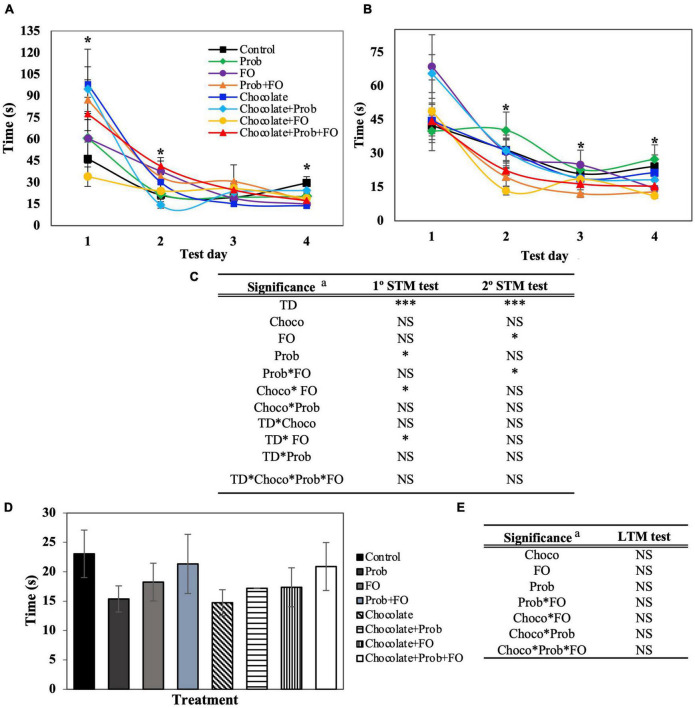
Total latency of rats using Barnes Maze test. STM, short-term memory; LTM, long-term memory; TD, test days; Choco, chocolate; FO, fish oil; Prob, probiotics; NS, non-significant. ^a^ Asterisks (*) indicate significant difference LSD test: *p < 0.05, ***p < 0.001. **(A)** First session of STM test, **(B)** Second session of STM test (after 45 min), **(C)** Significance of treatments interactions and test days on the first and second STM test, **(D)** LTM test (7th day), **(E)** Significance of treatments interactions and test days on the LTM test. Values are means (*n* = 8), and bars indicate the standard error of the mean.

To determine the effect of chocolate, ω3 PUFAs, and probiotics on the spatial learning and memory function in development rats, the BM test was performed to evaluate STM and LTM using different parameters. The total time (total latency) is shown in Figure 2, representing the time until the rat reached the scape hole or time was finished. Regarding the first and the second (after 45 min) BM test sessions, significant differences between treatments (*p* < 0.05) were shown in the STM test for both sessions (Figure 2).

In Figure 2A, an increase in the total latency time is observed in the first session of the first day in the chocolate treatment (98.00 ± 24.39 s) and chocolate + Prob (94.69 ± 15.46 s) treatment groups as compared to the control group (46.29 ± 11.70 s). Similarly to the first session, it was found that on day two, there were significant increases in the latency time for chocolate + Prob + FO treatments (41.30 ± 5.95 s) when compared to controls (21.30 ± 7.72 s). Until day four, a significant decrease was observed for chocolate (13.73 ± 1.54 s), FO (14.93 ± 2.98 s), Prob + FO (17.19 ± 4.37 s), chocolate + FO + Prob (17.26 ± 2.79 s), and chocolate + FO (18.99 ± 2.69 s) as compared to the control (29.53 ± 4.44 s).

On the other hand, during the second session carried out 45 min later (Figure 2B), a significant decrease in the total latency (*p* < 0.05) of the chocolate + FO treatment group was observed on day two (13.47 ± 1.97 s), and four (11.12 ± 0.87 s) compared to control 31.53 ± 4.74 s and 24.19 ± 5.1 s, respectively. Furthermore, no significant differences were observed for LTM (72 h later) analysis on the seventh day (Figure 2C). The latency time until the first exploration is shown in Supplementary Figure 1.

**FIGURE 3:**
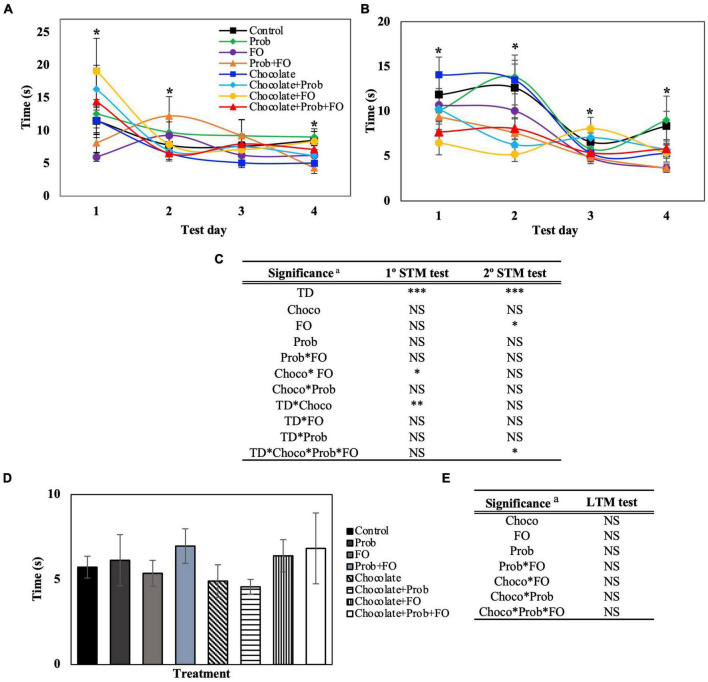
Latency in the escape zone of rats using the Barnes Maze test. STM, short-term memory; LTM, long-term memory; TD, test days; Choco, chocolate; FO, fish oil; Prob, probiotics; NS, non-significant. ^a^ Asterisks (*) indicate significant difference LSD test: *p < 0.05, **p < 0.01, ***p < 0.001. **(A)** First session of STM test, **(B)** Second session of STM test (after 45 min), **(C)** Significance of treatments interactions and test days on the first and second STM test, **(D)** LTM test (7th day), **(E)** Significance of treatment interactions and test days on the LTM test. Values are means (*n* = 8), and bars indicate the standard error of the mean.

**FIGURE 4:**
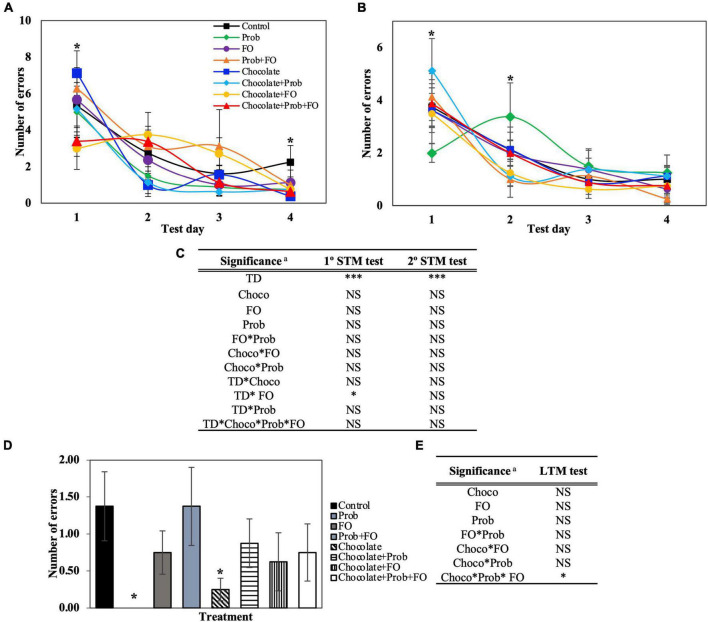
Total number of errors using the Barnes Maze test. STM, short-term memory; LTM, long-term memory; TD, test days; Choco, chocolate; FO, fish oil; Prob, probiotics; NS, non-significant. ^a^ Asterisks (*) indicate significant difference LSD test: *p < 0.05, ***p < 0.001. **(A)** First session of STM test, **(B)** Second session of STM test (after 45 min), **(C)** Significance of treatments interactions and test days on the first and second STM test, **(D)** LTM test (7th day), **(E)** Significance of treatment interactions and test days on the LTM test. Values are means, and bars indicate the standard error of the mean.

Data obtained from the BM test for the latency time (s) of the rats in the escape zone is shown in Figure 3. The scape zone is delimited by a 90° angle where the escape hole is in the middle, surrounded by two holes on each side. For this parameter, STM showed significant differences (*p* < 0.05) for the first and second sessions (Figures 2B, 3A). On the fourth day, in the first session (Figure 3A) Prob + FO (4.32 ± 0.85 s) treatment showed a decreasing latency time compared with the control group (8.50 ± 1.39). On the other hand, in the second session, there was a decreased latency time in the chocolate + FO groups compared to the control (12.63 ± 2.65 s) on the first two days with 6.47 ± 1.33 s and 5.20 ± 0.79 s, respectively. Also, on the third day of the second session, the treatment chocolate + FO (8.06 ± 1.27 s) showed a significant increase in time compared to the individual compounds FO (4.81 ± 0.64 s) and chocolate (5.33 ± 0.93 s). Also, the combination chocolate + FO (8.06 ± 1.27 s) showed a significant increase compared to the treatment Prob + FO (4.90 ± 0.54 s). Furthermore, significant differences were found on the fourth day of the second session (Figure 4B) for the FO (3.65 ± 0.46) and Prob + FO (3.65 ± 0.46) groups compared to the control (8.35 ± 1.66 s). No significant differences were found for LTM tests on the seventh day (Figure 3C). The latency of the first exploration in the escape hole (Primary latency) of rats using the Barnes Maze test is shown in Supplementary Figure 2.

#### Number of errors and distance traveled were affected by chocolate and fish oil ingestion in the rodent diet

The total number of errors (Figure 4) is another important parameter that evaluates the number of times a rat introduces their head into a hole, excluding the escape hole. The data obtained from the BM showed significant differences (*p* < 0.05) in some of the test days of STM and LTM tests (Figures 3B,C, 4A). On the first day of the first session, the chocolate + FO treatment decreased on time (3.00 ± 1.14 s) compared to the individual treatment chocolate (7.13 ± 1.22). On the fourth day of the first session (Figure 4A), a significant decrease in the average total errors in chocolate (0.38 ± 0.025) and chocolate + Prob + FO (0.63 ± 0.35) groups were found compared to the control (2.25 ± 0.91). In the second session, there was a significant increase in the average error of chocolate + Prob (5.13 ± 1.22) compared to Prob (2.00 ± 0.35). Inversely, by second session of day two, chocolate + Prob (1.13 ± 0.37) decreased compared to Prob (3.38 ± 1.29). Furthermore, for the long-term memory test (Figure 4C) Prob treatment did not show errors after 72 h, whereas the control showed 1.38 ± 0.47. Additionally, total errors decreased for the chocolate treatment to 0.25 ± 0.15 in the LTM test, being one of the lowest values on day four of the first session (0.38 ± 0.25) (Figure 4A). The number of errors in the escape zone, as well as the total distance traveled, are shown in the Supplementary Information (Supplementary Figures 3, 4).

The number of errors in the escape zone, shown in Supplementary Figure 4, complements the previous parameter (Figure 4). This parameter only includes the number of errors when rats inserted their head into any of the four holes around the escape hole at a 90° angle. For the STM analysis, just the first session (Supplementary Figure 3A) showed significant differences on the second and fourth days. No differences were found between groups compared to the control. LTM analysis showed no errors for the Prob treatment on the seventh day (Supplementary Figure 3C).

### Glucose and tissue analysis

Once all the behavioral analyses were finished, the rat’s brain and adipose tissue were dissected, and glucose was measured after euthanasia. Data obtained for all the groups are shown in Table 2. The results showed that the different chocolate formulations did not affect the variables. Blood sugar levels showed a significant increase in Prob (250.43 ± 17.67 mg/ml) treatment compared to the control (168.14 ± 17.03 mg/ml). Furthermore, encephalon weight analysis did not present significant differences between the groups and the control (*p* > 0.05). Finally, epididymal tissue weight analysis showed a significant increase (*p* < 0.05) in the chocolate (5.96 ± 0.28 g) treatment compared to the control (4.85 ± 0.26 g), being chocolate treatment with the highest value among the groups. Also, values of chocolate + Prob treatment showed a 17.62% decrease compared to chocolate alone. Furthermore, a 20.99% increase was observed for the chocolate + FO treatment compared to the FO group. In addition, chocolate + Prob + FO treatment presented a 22.99% decrease in epididymal tissue compared to chocolate alone.

**TABLE 2:** Effect of treatments in glucose concentration, encephalon, and epididymal weight after Barnes Maze test.

Treatment	Glucose^1^	Encephalon weight^2^	Epididymal tissue^2^
Control	168.14 ± 17.03 b	1.39 ± 0.02 a	4.85 ± 0.26 bc
Prob	250.43 ± 17.67 a	1.41 ± 0.02 a	4.83 ± 0.38 bc
FO	211.63 ± 26.63 ab	1.35 ± 0.03 a	4.33 ± 0.33 c
Prob + FO	181.75 ± 22.37 ab	1.39 ± 0.03 a	5.00 ± 0.28 bc
Chocolate	206.25 ± 18.14 ab	1.38 ± 0.02 a	5.96 ± 0.28 a
Chocolate + Prob	176.88 ± 27.50 ab	1.36 ± 0.02 a	4.91 ± 0.28 bc
Chocolate + FO	225.13 ± 33.25 ab	1.41 ± 0.03 a	5.48 ± 0.38 ab
Chocolate + Prob + FO	180.00 ± 24.94 ab	1.36 ± 0.04 a	4.59 ± 0.13 bc
**Significance^a^**			
Choco	NS	NS	NS
FO	NS	NS	NS
Prob	NS	NS	NS
FO*Prob	NS	NS	NS
Choco*FO	NS	NS	NS
Choco*Prob	NS	NS	NS
Choco*Prob*FO	NS	NS	NS

All data are presented as means ± SD; different letters indicate statistically significant differences between treatments by the LSD test (*p* < 0.05). Treatments: Control, Prob = Probiotics, FO = fish oil added at concentration 3.24%. Results were expressed in ^1^mg/dl and ^2^g.

### RT-PCR microbial quantification

The analysis of the effect on gut microbiota using RT-PCR quantification is shown in Table 3. No significant differences (*p* > 0.05) are shown for total bacteria analysis, *Lactobacillus* spp. and *Bifidobacteria* spp. However, for *Enterobacteria* spp. Analysis, Prob (3.18 ± 0.16 log/ml) and chocolate + Prob (3.26 ± 0.11 log/ml) treatment showed a significant decrease compared to the control (4.06 ± 0.29 log/ml).

**TABLE 3:** RT-PCR microbial quantification after Barnes Maze test.

Treatment	Total Bacteria	*Lactobacillus*	*Bifidobacteria*	*Enterobacteria*
Control	7.17 ± 0.08 a	3.51 ± 0.15 a	3.51 ± 0.16 a	4.06 ± 0.29 a
Prob	7.22 ± 0.11 a	3.47 ± 0.22 a	3.47 ± 0.18 a	3.18 ± 0.16 c
FO	7.25 ± 0.04 a	3.15 ± 0.06 a	3.15 ± 0.16 a	3.63 ± 0.35 abc
Prob + FO	7.36 ± 0.06 a	3.50 ± 0.07 a	3.50 ± 0.04 a	3.74 ± 0.08 abc
Chocolate	7.16 ± 0.19 a	3.26 ± 0.19 a	3.26 ± 0.26 a	4.00 ± 0.45 ab
Chocolate + Prob	7.24 ± 0.12 a	3.58 ± 0.14 a	3.58 ± 0.30 a	3.26 ± 0.11 bc
Chocolate + FO	7.27 ± 0.11 a	3.40 ± 0.17 a	3.40 ± 0.17 a	3.43 ± 0.12 abc
Chocolate + Prob + FO	7.03 ± 0.13 a	3.82 ± 0.17 a	3.82 ± 0.37 a	3.75 ± 0.22 abc
**Significance^a^**				
Choco	NS	NS	NS	NS
FO	NS	NS	NS	NS
Prob	NS	NS	NS	NS
FO*Prob	NS	NS	NS	*
Choco*FO	NS	NS	NS	NS
Choco*Prob	NS	NS	NS	NS
Choco*Prob*FO	NS	NS	NS	NS

All data are presented as means ± SD of log cell number per gram of fecal samples. Different letters indicate statistically significant differences between treatments by the LSD test (*p* < 0.05). Treatments: Control, Prob = Probiotics, FO = fish oil added at concentration 3.24%. Results were expressed in log/ml. ^a^Asterisks (*) indicate that main effects and interactions are significantly different by ANOVA: *p < 0.05.

### Neural morphological analysis in CA1 and CA3 regions of the hippocampus

#### Quantitative microscopic analysis

**FIGURE 5:**
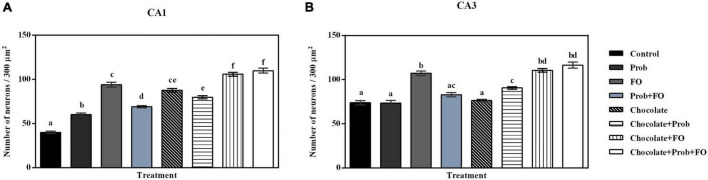
Nuclei count in the hippocampus of the different treatment brain samples. Prob, probiotics; FO, fish oil. **(A)** Hippocampal CA1 region, **(B)** Hippocampal CA3 region. Different letters among bars indicate statistically significant differences between treatments by the LSD test (*p* < 0.05).

Results from the analysis of the number of nuclei in each region of the selected hippocampus are shown in Figure 5. Significant differences were observed in the number of nuclei in the CA1 region. A significant increase in the number of nuclei was observed in the Chocolate + FO and chocolate + FO + Prob groups compared to the rest of the groups (*p* < 0.001). On the other hand, all the animals supplemented with the different treatments showed a more significant number of neuronal nuclei within the designated area compared to the control (*p* < 0.001) (Figure 5A). Regarding the analysis of the number of neurons in the CA3 region, significant differences were recorded. The comparison of means indicated a significant increase in the FO, chocolate + Prob, chocolate + FO, and chocolate + FO + Prob compared to the control group (*p* < 0.05). Likewise, the FO, chocolate + FO, and chocolate + FO + Prob groups had a higher number of neurons compared to the other treatments (*p* < 0.05). It should be noted that no significant differences were observed between the Prob, Prob + FO and chocolate groups concerning the control (*p* > 0.05) (Figure 5B).

**FIGURE 6:**
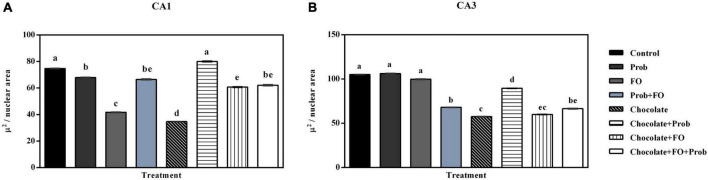
Nuclear area in neurons in the hippocampus of the different treatment brain samples. Prob, probiotics; FO, fish oil. **(A)** Hippocampal CA1 region, **(B)** Hippocampal CA3 region. Different letters among bars indicate statistically significant differences between treatments by the LSD test (*p* < 0.05).

Regarding the analysis of the nuclear area in the CA1 region, significant differences were found among groups (Figure 6). Except for the chocolate + Prob group, which was like the control (*p* > 0.05), it was observed that the rest of the groups showed a significant decrease in their area compared to the control (*p* < 0.05). The analysis also indicated a considerable reduction of this parameter in the FO and chocolate groups compared to the other treatments (*p* < 0.05), differences were also observed in the nuclear area of the Prob and chocolate + Prob + FO groups (*p* < 0.05) (Figure 6A). On the other hand, when comparing the nuclear area in neurons in the hippocampal CA3 region, significant differences were observed between the groups (*p* < 0.001); the *post-hoc* analysis showed a reduction in the nuclear area Prob + FO, chocolate, chocolate + Prob, chocolate + FO, and chocolate + Prob + FO compared to the control group (*p* < 0.05); the Prob and FO groups maintained a nuclear area similar to the control group (*p* > 0.05) (Figure 6B).

#### Histological description

**FIGURE 7:**
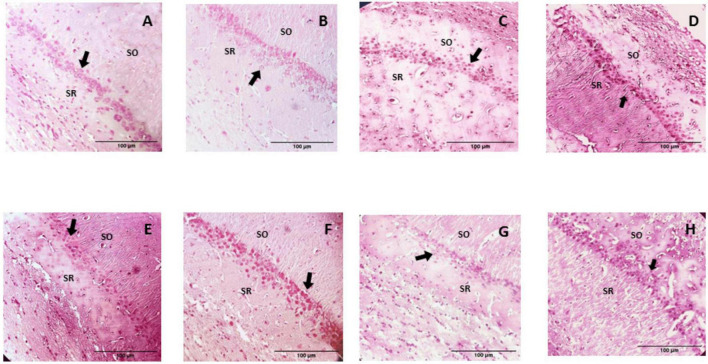
H-E Staining of the hippocampal CA1 region from study groups. Prob, probiotics; FO, fish oil; SO, stratum oriens; SR, stratum radiatum. **(A)** Control, **(B)** Prob, **(C)** FO, **(D)** Prob + FO, **(E)** chocolate, **(F)** chocolate + Prob, **(G)** chocolate + FO, **(H)** chocolate + FO + Prob. The main finding was the observation of apparently smaller nuclei in the pyramidal neurons of hippocampal CA1 region, particularly in the chocolate, chocolate + FO, and FO groups. Arrows point to neuronal nuclei. No damage or degenerative change was observed. Amplification 40x.

**FIGURE 8:**
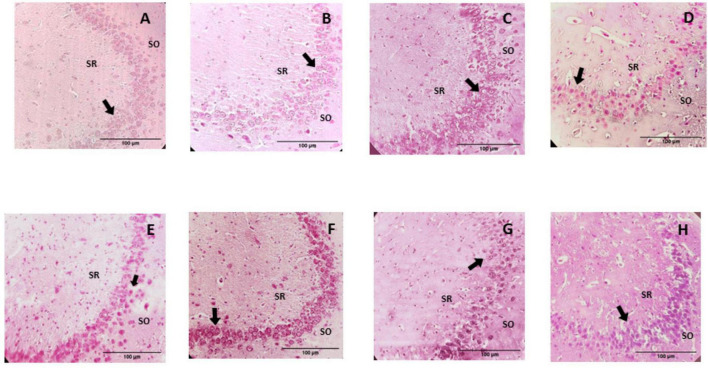
Photomicrographs of the hippocampal CA3 region from study groups. Prob, probiotics; FO, fish oil; SO, stratum oriens; SR, stratum radiatum. **(A)** Control, **(B)** Prob, **(C)** FO, **(D)** Prob + FO, **(E)** chocolate, **(F)** chocolate + Prob, **(G)** chocolate + FO, **(H)** chocolate + FO + Prob. The most important finding in the hippocampal CA3 region was the observation of apparently smaller nuclei in the pyramidal neurons, in addition to a slight basophilia, particularly in the chocolate, chocolate + FO, and FO groups. No damage or degenerative change was observed in any of the groups. Arrows point to neuronal nuclei. H-E Staining. Amplification 40x.

The microscopic analysis of the histological preparations in the coronal section did not indicate characteristics of degenerative damage in any of the groups. In this regard, profiles of almost spherical nuclei, clear, with granular chromatin and prominent nucleoli, were found in the CA1 and CA3 regions of the control group; the neuropil is observed to be uniform and intact (Figures 6, 7). While in the group of rats supplemented with pellets and probiotics, nuclei similar to those present in the control group were observed, although with slight basophilia in some nuclei of both regions (Figure 8). On the other hand, in the FO group, a reduction in the perimeter was described in most of the nuclei of the CA1 region as compared to the control, occasionally larger nuclei were observed interspersed; while in the CA3 region, the nuclei were slightly smaller in size than the control (medium) and greater basophilia was evident (Figure 8). The animals of the Prob + FO group showed a smaller size than the control group, with some larger nuclei interspersed in both regions. Similar observations were made in the group of animals supplemented exclusively with chocolate, where small nuclei were found with the intermittent presence of some nuclei similar to those described in the control. When the tissues of animals in the chocolate + Prob group were analyzed, good integrity of the neuropil was found, with a slight reduction in the perimeter in both regions. On the other hand, chocolate + FO, in addition to the diet, seems to generate predominantly similar to those already described in the chocolate + Prob group, with the presence of some medium-sized basophils in both regions. Likewise, in the chocolate + prob + FO group, medium-sized nuclei predominate and are similar to those described in the control group in both regions; in CA3, medium-sized nuclei are interspersed with large ones.

When comparing feed consumption during the weeks of treatment, no significant differences were found between the experimental groups in any of the weeks of treatment (Supplementary Figure 5).

## Discussion

### Spatial learning and memory evaluation

The current results from all the variables demonstrated a decrease-like tendency in the latency time for all groups. This shows that after some tests in the acquisition trials, all groups could carry out the learning task indicating no impairments in the STM and LTM (28), although there are variations between groups. All this data validates the potential use of chocolate as a vehicle to deliver a combination of bioactive ingredients (Prob + FO) to decrease total latency, primary latency, and latency until the first exploration. For the case of variables that showed a better performance when the bioactive ingredients were provided in chocolate, it could be inferred that other bioactive compounds present in chocolate (i.e., chocolate flavonoids) could also exert a positive response in the latency times. This observation could be attributed to the chocolate flavonoids (29). Previous studies have reported cognitive effects after consuming cocoa flavonoids, highlighting improving cognitive performance, specifically attention and working memory (30) and helping people with cognitive impairment (31). Milk chocolate’s flavonoid content and antioxidant capacity are similar to other flavonoid-rich products such as wine, black tea, and cranberry juice (32). Previous studies in monosodium glutamate (MSG)-induced obese aged rats as an experimental rodent model for Alzheimer’s disease (AD) showed a decrease in latency time when rodents were supplemented with dark chocolate (70% cocoa). A beneficial effect of antioxidants in dark chocolate has been demonstrated to enhance this model’s cognitive function (33).

In addition, the Prob + FO interaction showed a positive effect on cognitive performance in the BM test. Recent scientific reports confirmed the positive relation of ω3 PUFAs against inflammation and LPS-producing bacteria to promote gut microbiota regulation (34). The FO is an ingredient capable of generating a beneficial effect on memory, as demonstrated in this study. The ω3 PUFAs are structural components of the neuronal membranes and influence neuronal functions, including synapsis, influencing cognitive processes such as learning and memory (35). This is supported by various investigations where fish oil supplementation reduced locomotor hyperactivity, thus positively impacting anxiety-like behaviors, a reference parameter of spatial memory in the BM test (36). Therefore, the reduction of hyperactivity is reflected in decreased latency times to enter the escape hole. It was reported that DHA-enriched fish oil supplementation mitigated the diminished recognition memory induced by menopause in female humanized APOE4 mice carriers. Although the supplementation with DHA-rich fish oil did not improve the spatial working memory, it significantly affected the recognition memory, as demonstrated in the novel object recognition test. Additionally, fish oil supplementation improved the brain-derived neurotrophic factor levels, likely contributing to the improved cognitive function observed (37).

It is assumed that the individual’s capacity to locate the escape hole will have shorter latency times and fewer errors (38). The null number of errors found in the LTM test for Prob treatment and the significant decrease in the STM test of Prob + FO compared to the control showed that Prob treatment might improve memory in rats. The absence of errors for Prob means that the rodents could correctly remember the escape box location (38). It has previously been found that supplementation for four weeks with a mixture of probiotics has positively affected the cognition of subjects under acute stress (39). For example, the increase of probiotics by supplementing fermented milk with *L. plantarum* showed a significant decrease in latency time of rats, thus improving their learning ability (40). Also, it was demonstrated that *L. plantarum* PS128 alleviated 1-methyl-4-phenyl-1,2,3,6-tetrahydropyridine-induced neurodegeneration and neurotoxicity in a Parkinson’s disease mouse model (9). The oral gavage administration of PS128 increased the numbers of tyrosine hydroxylase-positive dopaminergic neurons in the substantia nigra and the striatal tyrosine hydroxylase expression level (9). In addition, it was reported that 21 days orally consumption of *L. rhamnosus*, *L. reuteri*, or *L. plantarum* significantly prevented LPS-induced neuroinflammation and memory decline, and specifically, *L. rhamnosus* prevents the downregulation of CaMKII-α, a serine/threonine protein kinase with a key role in long term potentiation, synaptic plasticity, learning and memory (41).

Also, in other animal models, supplementing probiotic strains such as *Pediococcus acidilactici* has previously decreased the number of birds’ errors compared to the control, thus improving memory. *Lactobacillus* and *Bifidobacterium* strains used individually or in combination have been proved to enhance alterations in spatial and consolidated memory (42). Furthermore, the supplementation of an adequate ω3 PUFAs was found to decrease the total of errors compared to a ω3 PUFAs deficient diet. This decrease may be due to rats’ capacity to learn to reduce the number of perseverations or repeated visits to other adjacent holes. This increase in errors suggests that the deficiency of ω3 PUFAs may suppress previous learnings, causing a deficit in behavior flexibility, thus impairing rat performance (43). Considering the individual effects of Prob and FO, both may improve the total of errors that reflect on memory performance. Here, the reduction of the number of errors of chocolate + Prob + FO treatment compared to the control may be due to the interaction of chocolate in synergy with FO and Prob.

The rat’s capacity to remember the escape hole location can also be measured by the time elapsed and interaction with the holes in the target quadrant, known as the escape zone. In this case, a rodent with an intact memory would spend more than 25% of the time elapsed in the escape zone (38). Regarding the latency time in the escape zone (Figure 3), chocolate + FO showed an increase in the percentage of time (56.2% 1st and 44% 2nd STM test). Chocolate + Prob + FO treatment showed an increase (37.8%) on the fourth day of the 2nd STM test. Both groups, chocolate + FO and chocolate + Prob + FO showed the highest values in the LTM test (36.8 and 32.9%). Control of the LTM test showed a percentage of 24.9%. Considering this information, both chocolate + FO and chocolate + Prob + FO groups showed an intact memory because percentages were higher than 25%. Control treatment behaves within the limit, but this statement highlighted that combining the three ingredients improved memory and learning.

On the other hand, Prob treatment showed a high percentage of 39.9% on the LTM test, showing that probiotics supplementation may improve long-term memory. Previous studies showed supplementation of probiotic mix (*L. acidophilus* CUL60, *L. acidophilus* CUL21, *B. bifidum* CUL20, and *Bifidobacterium lactis* CUL34) generates a positive impact on long-term potentiation, associated with long-term synaptic plasticity. Probiotic mix was found to positively impact learning in an AD rat model, by releasing neurotransmitters *via* pre-synaptic mechanisms or by protecting serum lipid profiles (44). Interestingly, the effect of probiotics on cognitive function across the human lifespan has been explored. The systematic review of 30 experimental studies investigating the effects of probiotics on different ages and cognitive statuses suggests that probiotics may enhance cognitive function in adult populations with some cognitive impairment (45). The administration of 10^8^ CFU/ml of *Limosilactobacillus fermentum* improved the cognitive processes of learning, memory, language, and visuospatial function in people older than 60 with mild cognitive impairment (46).

Surprisingly, no error in the escape zone was found for the Prob treatment in the LTM because there were no accumulated errors. The effect of Prob, in this case, is the same as shown above for the total number of errors, where probiotic strains have been found to improve memory and spatial learning. On the other hand, chocolate + Prob + FO treatment showed an increase in the escape zone errors compared to chocolate + Prob treatment and Prob. This can be translated into that chocolate, and FO generated a beneficial effect by committing the errors within the escape zone, meaning that the memory of the rats in the chocolate + Prob + FO treatment improved (38).

### Glucose and tissue analysis

Abnormalities in the glucose regulation, particularly high levels, are negatively associated with cognitive function and changes in the brain structure, including brain atrophy (47, 48). This study showed no differences in blood sugar, body weight, and brain weight among groups supplemented with chocolate. Since probiotic strains, such as *L. plantarum* and *L. rhamnosus*, have been found to regulate blood sugar levels (49, 50), the blood sugar increase of Prob treatment should be further studied since it contradicts previous reports. Recent studies showed that stress could be associated with increased fasting glucose (51). Furthermore, the chocolate + Prob + FO treatment did not present a significant increase (*p* > 0.05) in glucose compared to the control treatment, indicating that chocolate added with fish oil and probiotics did not modify the blood glucose parameters. Therefore, the cognitive differences between the different groups, and described in the previous section, can be attributed to the components of the supplemented chocolate.

As mentioned above, the brain weight loss (grey matter) due to neuronal cell death, results in cognitive impairment (52, 53). However, in this study, any group did not show a change in brain weight compared with the control group. This normal brain weight confirms the cognitive health of the rats.

Finally, epididymal tissue is an active metabolically fat depot used widely to study rodent adipose tissue. Epididymal fat attaches to the testis and epididymis. Recent investigations showed that epididymal fat is higher in obese mice. In this model, epididymal fat is correlated with body weight (54). The slight weight increment shown for chocolate compared to the control may be partially caused by the fatty acid content in cocoa butter that may contribute to the rapid expansion of the tissue (55). Otherwise, adding Prob into the chocolate vehicle helped to decrease tissue weight gain compared to the chocolate alone. This decrease may be attributed to the interaction of both probiotic strains added. It has been found that *L. plantarum* strains decreased epididymal fat weight in high-fat diets. Also, *L. rhamnosus* GG significantly attenuated obesity markers and macrophage infiltration into epididymal tissue (56). Since no significant changes were presented between the chocolate + Prob + FO and the control group, we conclude that the milk chocolate can be used to deliver probiotic and ω3 PUFAs with a significant impact on the cognitive function and without the detrimental effects of fat accumulation and high glucose serum levels.

### RT-PCR microbial quantification

The concept of the brain-gut axis as a bidirectional channel of information exchange between the brain and the sympathetic and parasympathetic nervous system has been associated with good cognition and pathological processes such as AD and Parkinson’s disease. Poor diets have been associated with lower microbiota diversity, inflammation, and disability. Thus, suffering a neurodegenerative disorder by a nutritional deficiency and dysbiosis is very likely (57, 58). Although this brain-gut information exchange mechanism is still unclear, increasing evidence indicates that the gastrointestinal microbiota is a key player in the cognitive functions (58). Interestingly, the manipulation of gut microbiota could be a promising avenue for enhancing cognitive function (59).

Microbial strains such as *Lactobacillus* and *Bifidobacterium* have been reported as beneficial probiotic strains (60). In this case, no significant changes were shown for any groups, including chocolate + Prob + FO, which means that neither the individual components nor the vehicles used negatively affect these probiotic strains. Cognitive decline in the senescence aging process has been related to gut microbiota imbalances such as dysbiosis (61). On the other hand, proinflammatory bacteria such as *Enterobacteria* were also found as indicators of dysbiosis (61). This altered gut bacterial composition has been associated with inflammatory diseases and long-term neurological outcomes (62). In this study, there is a significant decrease of *Enterobacteria* spp. in the Prob and chocolate + Prob groups, which indicates that both treatments can prevent dysbiosis. As previously reported, this is probably mediated by *L. plantarum* (40, 63). Also, it was reported that *L. plantarum* treatment increases the colonic microbiota diversity and Firmicutes/Bacteroidetes ratio but a reduced relative abundance of *Lactobacillus* (64). In another study, *L. plantarum* NA136- boosted the population density of *Allobaculum*, *Lactobacillus*, *Bifidobacterium*, *Helicobacter*, *Barnesiella*, *Parabacteroides*, and *Parasutterella*, which produce short-chain fatty acids that strengthen the intestinal barrier, modify the intestinal permeability and LPS production levels, and competitively inhibit pathogenic bacterial growth (65). More extended studies are required to evaluate the effects of chocolate + Prob formulation on microbiota diversity.

### Neural morphological analysis in CA1 and CA3 regions of the hippocampus

This study investigated the effect of the consumption of milk chocolate added with probiotics FO and ω3 PUFAs on the cytoarchitectural integrity of the hippocampus, particularly of the CA1 and CA3 subregions. Likewise, morphometric parameters such as the number of neurons included in a surface and the nuclear area of the neurons in these subregions were evaluated. Among the most outstanding findings, there is no evidence of degenerative damage in the analyzed surfaces of the hippocampus. There was evidence of a marked increase in the number of neurons in both hippocampal regions, in groups chocolate + Prob + FO and chocolate + FO. A smaller area was quantified in the nuclei of neurons in both hippocampal subregions in these same groups. Interestingly, these results are in agreement with those described in section “Cognitive evaluation” were the groups with a greater number of neurons, are the ones with the best outcomes in the cognitive evaluation. For example, administration of chocolate + FO treatment, significatively reduced the total latency time during several days (first, 4th day; second session, 2nd and 4th day), reducing number of errors (first session, 4th day) and increasing latency time (second session, 3rd and 4th day). Also, administration of chocolate + FO + Prob, showed a significant decrease in latency time (first session, 4th day) and number of errors (first session, 4th day). Thus, the best performance in the BM can be associated with the increased neuron density, and probably with a higher neuroplasticity, which in turn, can enhance the learning and memory processes in the rat.

The hippocampus is part of the limbic system and has important implications for learning and memory functions and emotional response systems (18, 66). The hippocampal formation is made up of the hippocampus proper [Cornus Ammonis (CA) 1-4] and dentate gyrus (DG), the subiculum, and the entorhinal cortex (67). Regarding its cytoarchitecture, most of the hippocampal neurons are of the glutamatergic type, which means that they are excitatory, including the great pyramidal neurons of the Horn of Ammon and the granule neurons of the dentate gyrus. The rest of the cells are non-pyramidal GABAergic inhibitory interneuron neurons of diverse subpopulations (68, 69). Evidence has shown that changes in the cytological characteristics of the hippocampal subregions may be related to modifications in cognitive functionality (70, 71). However, results from this study demonstrated no adverse effects associated with the consumption of the different treatments. One could even highlight the existence of a positive impact after consuming chocolate + FO. Studies have shown an increase in the number of hippocampal neurons due to treatment with ω3 PUFAs through neurogenesis (72). Adult neurogenesis has been widely associated with neurophysiologic processes such as motor function, learning, and memory (73). Although the effect of a dietary compound on neurogenesis is not fully clear, the evidence demonstrated that bioactive substances such as ω3 PUFAs could modulate the brain structure and function, thus, modulating cognitive ability (74).

In this sense, the increased number of neurons per area in the groups mentioned above may be directly related to neurogenesis in the subregions analyzed. On the other hand, the nuclear area has been classically considered an indicator of the cells’ metabolic, transcriptional, and functional activity (75, 76). In this context, the decrease in the nuclear area shown by a good number of neurons could be explained by the significant presence of *non-pyramidal-type* cells in the CA1 and CA3 regions. This would contrast with the idea that a decrease in the nuclear area could indicate a downward functional trait of these neurons (77) and be associated with the different treatments applied; however, there is no evidence of degenerative damage. Here we found a marked increase in the number of neurons in the chocolate + Prob + FO and chocolate + FO groups, which correlates with the enhanced spatial cognitive function in BM. Impairments in the hippocampus-dependent learning and memory could be associated with reduced neurogenesis and synaptic alterations in the hippocampal formation (78, 79). In agreement with this data, Giacomini et al. (80), reported that oil corn increased the number of neurons in the hippocampus, while the control group had a reduced number of granular neurons in a Down syndrome mouse model. They argued that the latency reduction in the Morris water maze indicates an improvement in spatial learning, consistent with the restored hippocampal neurogenesis and dendritogenesis exhibited after corn oil treatment in the Down syndrome mouse model (80). Similarly, Jansen et al. (81) demonstrated a beneficial correlation between dietary ω3 PUFAs and significant effects on motor coordination, exploratory behavior, sensorimotor integration, spatial memory, and neurogenesis (81). Altogether, our findings demonstrated that chocolate + Prob + FO positively affects brain functions and cognitive performance. However, more investigations are required to warrant clinical significance and verify their roles as therapeutical tools.

## Conclusion

In the present study, it was demonstrated that probiotics and fish oil, either alone or combined, improve spatial learning and memory in male Wistar rats. Moreover, the biological activity of the tested ingredients was retained and, in some instances, improved when chocolate was used as the delivery system. Further experiments should be conducted to better understand the role of chocolate in improving cognition. Moreover, the positive effect of the formulation on cognition should be validated in children in further clinical studies.

## Data availability statement

The original contributions presented in this study are included in the article/Supplementary material, further inquiries can be directed to the corresponding author.

## Ethics statement

This animal study was reviewed and approved by Bioethical Committee of University of Guadalajara (CINV/034/2022).

## Author contributions

PF-B, DJ-V, and JB-P: conceptualization. PF-B, DJ-V, AS, CH-B, EP-C, JB-P, and LA-L: methodologyand writing – review and editing PF-B and LA-L: formal analysis. PF-B and DJ-V: investigation. AS, EP-C, DJ-V, and CH-B: resources. PF-B, DJ-V, and ER-Z: writing – original draft preparation. DJ-V and JB-P: supervision. DJ-V, CH-B, AS, and JB-P: funding acquisition. All authors have read and agreed to the published version of the manuscript.
