# Supplementary material for: Fish oil and probiotics supplementation through milk chocolate improves spatial learning and memory in male Wistar rats
Source: Front Nutr. 2022 Nov 17;9:1023653. doi: 10.3389/fnut.2022.1023653 (PMC9712752; doi:10.3389/fnut.2022.1023653)
Supplement: Supplementary file 1 [file Data_Sheet_1.pdf]

Supplementary Material

Fish oil and probiotics supplementation through milk chocolate improves spatial learning and memory in male Wistar rats

Paulinna Faccinetto-Beltrán[1,2], Luis Octavio Aguirre-López[3], Jacinto Bañuelos-Pineda[3], Edwin E. Reza-Zaldívar[1], Arlette Santacruz[4], Carmen Hernández-Brenes[4,5], Esther Pérez-Carrillo[4], Daniel A. Jacobo-Velázquez[1,2]*

[1] Tecnologico de Monterrey, The Institute for Obesity Research, Av. General Ramón Corona 2514, Zapopan 45201, Jal, México
[2] Tecnologico de Monterrey, Escuela de Ingeniería y Ciencias, Av. General Ramón Corona 2514, Zapopan 45201, México
[3] Laboratorio de Morfofisiología, Departamento de Medicina Veterinaria, Centro Universitario de Ciencias Biológicas y Agropecuarias, Universidad de Guadalajara, Zapopan, México.
[4] Tecnologico de Monterrey, Escuela de Ingeniería y Ciencias, Av. Eugenio Garza Sada 2501, Monterrey 64849, México
[5] Tecnologico de Monterrey, The Institute for Obesity Research, Av. Eugenio Garza Sada 2501, Monterrey 64849, México

Supplementary Table 1. Quantitative real-time PCR (qPCR) primers.

Bacterial group	Primers sequences	Annealing temperature (ºC)	Fragment (bp)
Total Bacteria (From *E. coli*)	Fw 5’-TGGCTCAGGACGAACGCTGGCGGC-3’<br>Rv 5’-CCTACTGCTGCCTCCCGTAGGAGT-3’	61	339/ 539
Lactobacillus	Fw 5’-GGAAACAGRTGCTAATACCG-3’<br>Rv 5’-CACCGCTACACATGGAG-3’	61	340
Bifidobacteria	Fw 5’-CTCCTGGAAACGGGTGG-3’<br>Rv 5’-GGTGTTCTTCCCGATATCTACA-3’	55	549/ 563
Enterobacterium (From *E. coli*)	Fw 5’-CATTGACGTTACCCGCAGAAGAAGC-3’<br>Rv 5’-CTCTACGAGACTCAAGCTTGC-3’	62	340

The latency time (s) of the first exploration (Supplementary Figure 1) represents the time that elapses until the rat interacts for the first time with any of the holes, excluding the escape hole. Significant differences (p<0.05) were shown in STM and LTM tests (Supplementary Figure 1A, 1B, 1C). In session one of the STM tests, there were significant differences between groups for test days two and four (Supplementary Figure 1A). On session one and test day two, the combination of chocolate+FO (6.95±1.31 s) showed a significant decrease in time compared to the individual treatments of chocolate (18.07±5.01 s) and FO (19.21±5.45 s). However, none of these groups showed significant differences compared to the control (10.80±2.69 s). Furthermore, for the second session of STM tests (Supplementary Figure 1B), significant differences were shown for the first three days. On the first day, there was a significant increase in the exploration time in Prob (21.72±6.83 s) and chocolate+Prob (20.55± 4.19 s) groups compared to the control group (9.20±0.87 s). On the second day, the chocolate+Prob (18.40±4.69 s) treatment increased exploration time compared to the control

(8.05±1.17 s). On the other hand, FO treatment on day three showed a significant decrease (6.42±1.52 s, p<0.05) in exploration time compared with 17.08±4.03 s of the control treatment. Finally, in the LTM test, there is a decreasing exploration time in the Prob+FO (6.42±1.22 s) treatment compared to the control (12.36±2.39 s).

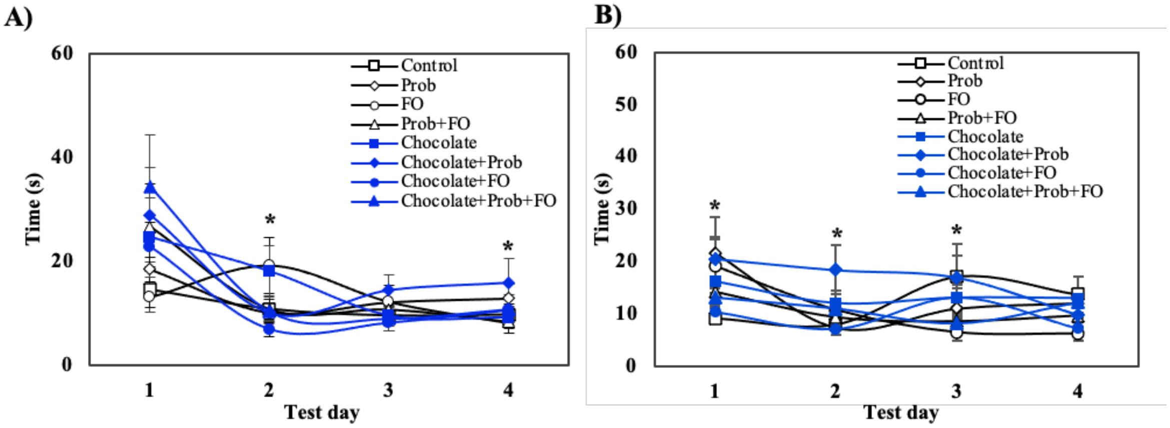

C)

Significance [a]	1° STM test	2° STM test
TD	***	**
Choco	NS	NS
FO	NS	**
Prob	NS	NS
Prob*FO	NS	NS
Choco*FO	NS	NS
Choco*Prob	NS	NS
TD*Choco	*	NS
TD*FO	NS	NS
TD*Prob	*	NS
TD*Choco*Prob*FO	NS	NS

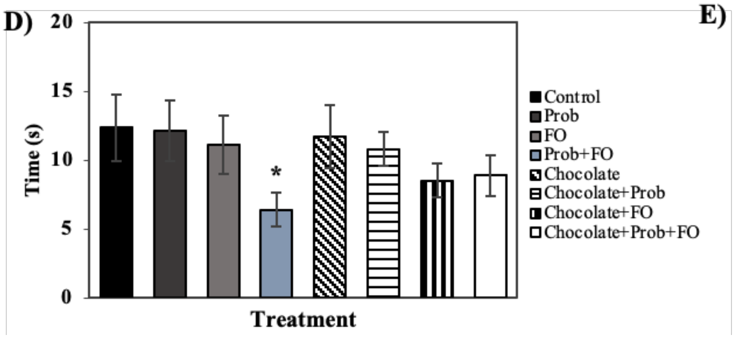

E)

Significance [a]	LTM test
Choco	NS
FO	NS
Prob	NS
Prob*FO	*
Choco*FO	NS
Choco*Prob	NS
Choco*Prob*FO	NS

Supplementary Figure 1. Latency until the first exploration of rats using the Barnes Maze test. Abbreviations: STM= short-term memory, LTM= long-term memory, TD= test days, Choco= chocolate, FO= fish oil, Prob= probiotics, NS= non-significant. [a] Asterisks (*) indicate significant difference LSD test (p<0.05): *p < 0.05, **p < 0.01, ***p < 0.001. A) First session of STM test, B) Second session of STM test (after 45 min), C) Significance of treatments interactions and test days on the first and second STM test, D) LTM test (7th day). E) Significance of treatment interactions and test days on the LTM test. Values are means, and bars indicate the standard error of the mean.

Another parameter evaluated with the BM memory test was the latency time (s) of the first exploration in the escape hole (Supplementary Figure 2). Significant differences were found on days one, two, and four of the first session (Supplementary Figure 2A). On the first day, data showed an increase in the time of Prob+FO (84.84±13.48 s), chocolate (94.62±25.00 s), and chocolate+Prob

(90.11± 14.83s) groups compared to the control (27.46±9.04 s). Later, on the second day, the lowest and most significant values were found in the chocolate+Prob (14.86±3.39 s) treatment compared with the control (40.67±14.57 s). Moreover, on the fourth day, the chocolate treatment decreased to 10.94±1.42 s compared to the control (25.70±4.37s). On the other hand, the second session showed significant differences from day two to four (Supplementary Figure 2B). Also, chocolate+FO treatment showed a significant decrease ($p<0.05$) on day two (11.31±1.91 s) and four (8.17 ±1.17 s) compared to their controls, 28.28±4.54 s and 20.05±4.8 s, respectively. No significant differences were found between the treatments for the latency of the first exploration in the escape hole in the LTM tests (Supplementary Figure 2D and 2E).

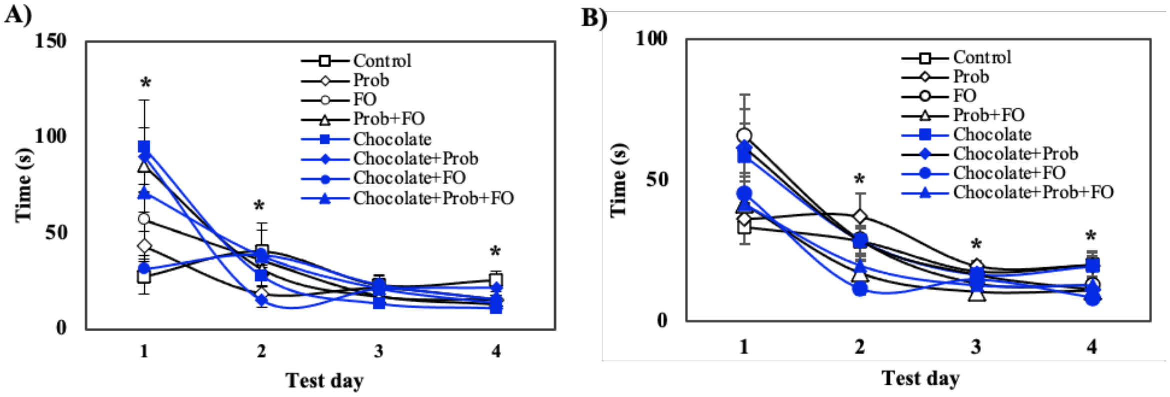

C)

Significance [a]	1° STM test	2° STM test
TD	***	***
Choco	NS	NS
FO	NS	*
Prob	NS	NS
FO*Prob	NS	NS
Choco* FO	*	NS
Choco*Prob	NS	NS
TD*Choco	NS	NS
TD*FO	NS	NS
TD*Prob	*	NS
TD*Choco*Prob*FO	NS	NS

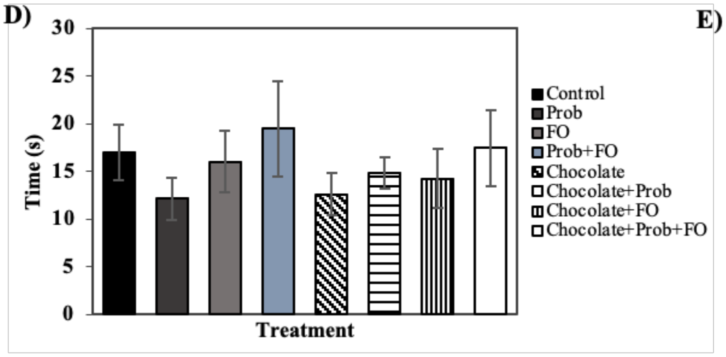

E)

Significance [a]	LTM test
Choco	NS
FO	NS
Prob	NS
FO*Prob	NS
Choco*FO	NS
Choco*Prob	NS
Choco*Prob*FO	NS

Supplementary Figure 2. The latency of the first exploration in the escape hole (Primary latency) of rats using the Barnes Maze test. Abbreviations: STM= short-term memory, LTM= long-term memory, TD= test days, Choco= chocolate, FO= fish oil, Prob= probiotics, NS= non-significant. [a] Asterisks (*) indicate significant difference LSD test ($p<0.05$): *$p < 0.05$, **$p < 0.01$, ***$p < 0.001$. A) First session of STM test, B) Second session of STM test (after 45 min), C) Significance of treatments interactions and test days on the first and second STM test, D) LTM test (7th day), E) Significance of treatment interactions and test days on the LTM test. Values are means (n = 8), and bars indicate the standard error of the mean.

The number of errors in the escape zone is shown in Supplementary Figure 3. This parameter only includes the number of errors when rats inserted their head into any of the four holes around the escape hole at a 90º angle. For the STM analysis, just the first session (Supplementary Figure 3A) showed significant differences on the second and fourth days. No differences were found between groups compared to the control. LTM analysis showed no errors for the Prob treatment on the seventh day (Supplementary Figure 3C).

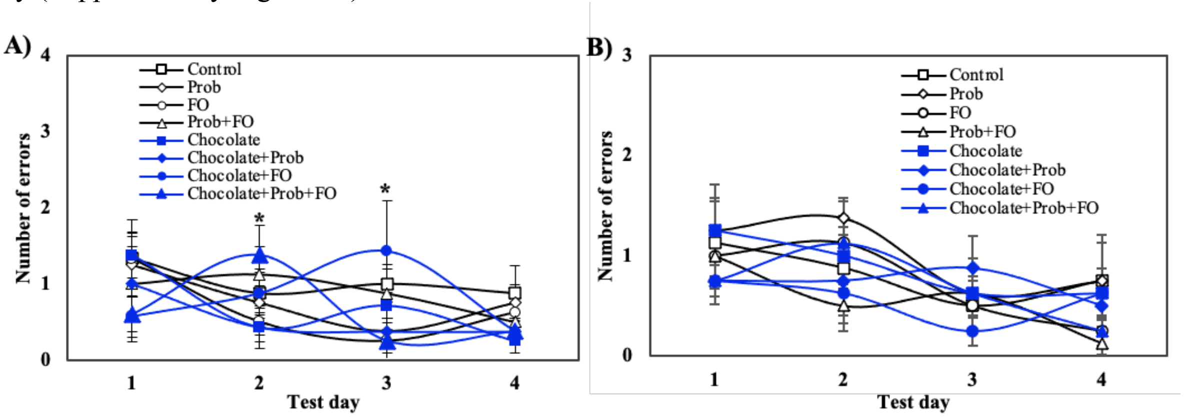

C)

Significance [a]	1º STM test	2º STM test
TD	*	*
Choco	NS	NS
FO	NS	NS
Prob	NS	NS
FO*Prob	NS	NS
Choco*FO	NS	NS
Choco*Prob	NS	NS
TD*Choco	NS	NS
TD*FO	NS	NS
TD*Prob	NS	NS
TD*Choco*Prob*FO	NS	NS

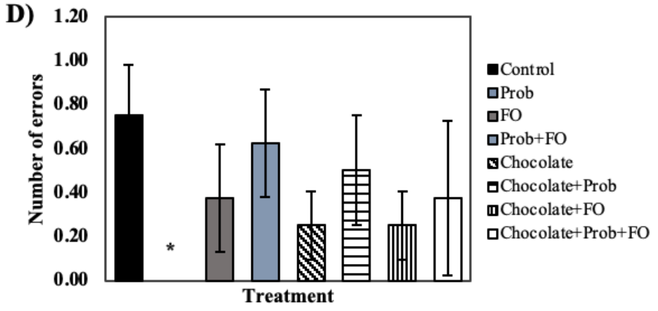

E)

Significance [a]	LTM test
Choco	NS
FO	NS
Prob	NS
FO*Prob	NS
Choco*FO	NS
Choco*Prob	NS
Choco*Prob*FO	NS

Supplementary Figure 3. Number of errors in the escape zone using the Barnes Maze test. Abbreviations: STM= short-term memory, LTM= long-term memory, TD= test days, Choco= chocolate, FO= fish oil, Prob= probiotics, NS= non-significant. [a] Asterisks (*) indicate significant difference LSD test (p<0.05): *p < 0.05, **p < 0.01, ***p < 0.001. A) First session of STM test, B) Second session of STM test (after 45 min), C) Significance of treatments interactions and test days on the first and second STM test, D) LTM test (7th day), E) Significance of treatment interactions and test days on the LTM test. Values are means (n = 8), and bars indicate the standard error of the mean.

The last parameter evaluated in the BM test was the total distance traveled by the rats (Supplementary Figure 4). Significant differences are shown for STM tests' first and second sessions (Supplementary Figure 4A and Supplementary Figure 4B). On the first session of the fourth day of STM analysis (Supplementary Figure 4A), a significant decrease in distance traveled (p<0.05) was

shown in FO (81.75±16.39 cm), chocolate (85.33±10.51 cm), and chocolate+Prob (99.51±17.63 cm) group compared to the control (181.52±29.80 cm). Furthermore, on the second day of the second session analysis (Supplementary Figure 4B), Prob+FO (82.53±4.54 cm) also decreased the distance traveled compared to the control (180.87±32.22 cm). No significant differences were found in the LTM test (Supplementary Figure 4C).

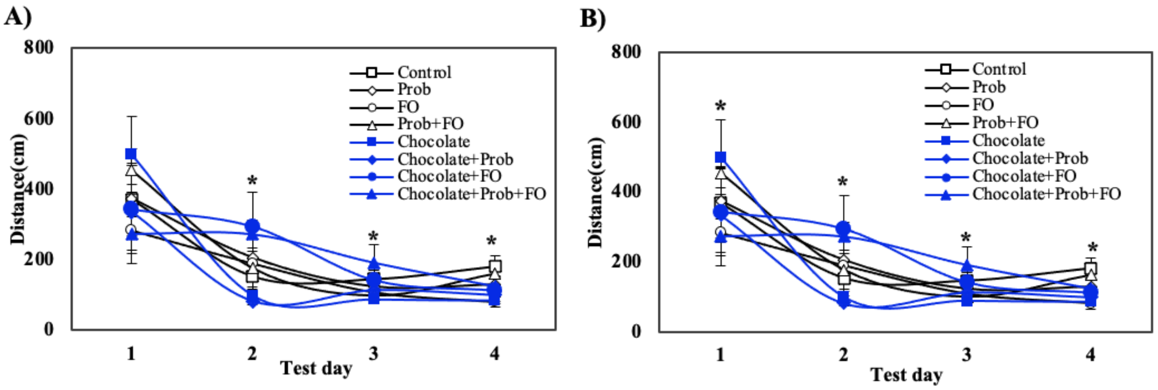

C)

Significance [a]	1° STM test	2° STM test
TD	***	***
Choco	NS	NS
FO	NS	NS
Prob	NS	NS
FO*Prob	NS	NS
Choco*FO	NS	NS
Choco*Prob	NS	**
TD*Choco	NS	NS
TD*FO	NS	NS
TD*Prob	NS	NS
TD*Choco*Prob*FO	NS	NS

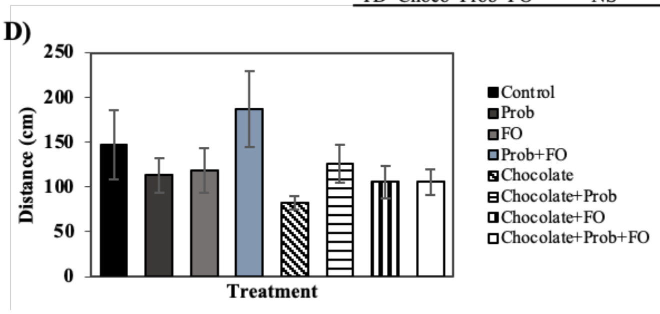

E)

Significance [a]	LTM test
Choco	NS
FO	NS
Prob	NS
FO*Prob	NS
Choco*FO	NS
Choco*Prob	NS
Choco*Prob*FO	NS

Supplementary Figure 4. Total distance traveled (cm) using the Barnes Maze test. Abbreviations: STM= short-term memory, LTM= long-term memory, TD= test days, Choco= chocolate, FO= fish oil, Prob= probiotics, NS= non-significant. [a] Asterisks (*) indicate significant difference LSD test (p<0.05): *p < 0.05, **p < 0.01, ***p < 0.001. A) First session of STM test, B) Second session of STM test (after 45 min), C) Significance of treatments interactions and test days on the first and second STM test, D) LTM test (7th day), E) Significance of treatments interactions and test days on the LTM test. Values are means (n = 8), and bars indicate the standard error of the mean.

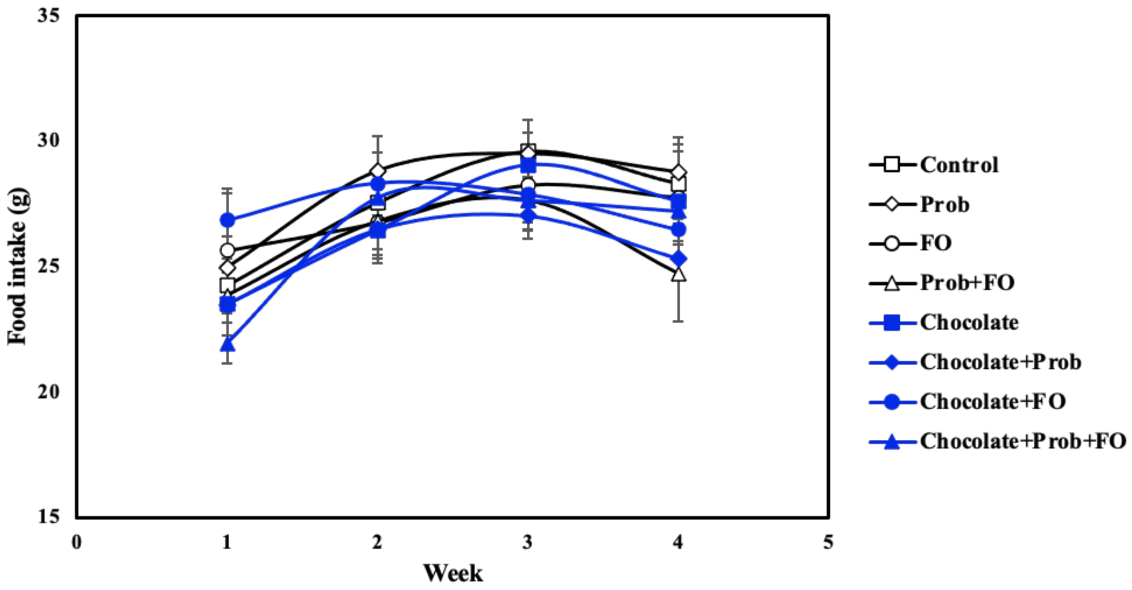

Supplementary Figure 5. Conventional food consumption during the treatment period (g). Food consumption of rats during a 4-week treatment period.
